# Taking shared decision making for prostate cancer to the next level: Requirements for a Dutch treatment decision aid with personalized risks on side effects

**DOI:** 10.1016/j.invent.2023.100606

**Published:** 2023-02-01

**Authors:** Laura M.J. Hochstenbach, Domino Determann, Rianne R.R. Fijten, Esther J. Bloemen-van Gurp, Renée Verwey

**Affiliations:** a Center of Expertise for Innovative Care and Technology (EIZT), School of Nursing, Zuyd University of Applied Sciences, P.O. Box 550, 6400 AN Heerlen, the Netherlands; b Department of Health Services Research, Care and Public Health Research Institute (CAPHRI), Faculty of Health Medicine and Life Sciences, Maastricht University, P.O. Box 616, 6200 MD Maastricht, the Netherlands; c PATIENT+, Zeestraat 76, 2518 AD Den Haag, the Netherlands; d Department of Radiation Oncology (MAASTRO), GROW School for Oncology and Developmental Biology, Maastricht University Medical Center^+^, P.O. Box 616, 6200 MD Maastricht, the Netherlands; e Expertise Center Empowering Healthy Behavior, Fontys University of Applied Sciences, P.O. Box 347, 5600 AH Eindhoven, the Netherlands

**Keywords:** Prostate cancer, Shared decision making, Patient decision aid, Personalized medicine, User centered design

## Abstract

**Background:**

Different curative treatment modalities need to be considered in case of localized prostate cancer, all comparable in terms of survival and recurrence though different in side effects. To better inform patients and support shared decision making, the development of a web-based patient decision aid including personalized risk information was proposed. This paper reports on requirements in terms of content of information, visualization of risk profiles, and use in practice.

**Methods:**

Based on a Dutch 10-step guide about the setup of a decision aid next to a practice guideline, an iterative and co-creative design process was followed. In collaboration with various groups of experts (health professionals, usability and linguistic experts, patients and the general public), research and development activities were continuously alternated.

**Results:**

Content requirements focused on presenting information only about conventional treatments and main side effects; based on risk group; and including clear explanations about personalized risks. Visual requirements involved presenting general and personalized risks separately; through bar charts or icon arrays; and along with numbers or words, and legends. Organizational requirements included integration into local clinical pathways; agreement about information input and output; and focus on patients' numeracy and graph literacy skills.

**Conclusions:**

The iterative and co-creative development process was challenging, though extremely valuable. The translation of requirements resulted in a decision aid about four conventional treatment options, including general or personalized risks for erection, urinary and intestinal problems that are communicated with icon arrays and numbers. Future implementation and validation studies need to inform about use and value in practice.

## Highlights

• Using CPMs to personalize outcomes are a next step in optimizing PDAs for localized prostate cancer.
• We developed a PDA including information about four conventional treatments and three main categories of side effects.
• Icon arrays, numbers and verbal explanation were used to communicate general and personalized risks of side effects.
• Co-creation with various experts intended to ensure optimal alignment with potential users and the situation in practice.

## 1 Introduction

The most common curative treatments for patients diagnosed with localized prostate cancer are radical prostatectomy (RP), external beam radiation therapy (EBRT), brachytherapy (BT) and active surveillance (AS). Given the lack of evidence for the superiority of one of these treatments in terms of survival and recurrence, current practice guidelines offer multiple options (Netherlands Comprehensive Cancer Organization; Sanda et al., 2018). These options are different in potential side effects, in terms of risk as well as severity and nature, making a treatment decision very personal. In order to make an informed and deliberate treatment choice, patients facing localized prostate cancer need to be given the right information. Practice however shows that, prior to choosing treatment, two-thirds of localized prostate cancer patients poorly understands differences in treatment risks (van Stam et al., 2018) and one third reports to be dissatisfied with the provided information (Lamers et al., 2016).

To better inform patients and subsequently improve shared decision making (SDM), the use of patient decision aids (PDAs) in clinical practice has proven to be beneficial (Adsul et al., 2015; Lin et al., 2009; Stiggelbout et al., 2012). Patients using a PDA generally feel better informed, have more accurate risk perceptions and experience less decisional regret (Stacey et al., 2017). Unfortunately, a systematic review of Violette et al. (2015) focusing on prostate cancer PDAs showed inconsistent effects on decisional outcomes and no effect on treatment decision. To some extent these findings might be due to shortcomings in communicative aspects, such as presenting statistical information without visual aids, including biased cross tables to compare treatment options, and lacking interactive methods to elicit values and preferences (Vromans et al., 2019). During the development of PDAs attention needs to be given to the best possible way to communicate about risks of different treatment options (Garcia-Retamero and Cokely, 2017), for which a user centered design is recommended. Apart from that, developers need to ensure PDA quality by applying the International Patient Decision Aids Standards (IPDAS) criteria (Elwyn et al., 2009).

Given the developments in personalized medicine and healthcare, using clinical prediction models (CPMs) - i.e. statistical algorithms that utilize patient, disease and treatment characteristics to estimate individual probabilities of health outcomes - are a next step in optimizing PDAs. To benefit from personalized risk information, patients should be educated about the nature of medical knowledge and the value of personalized risk information (Han et al., 2013). Based on their findings in a randomized controlled trial for prostate cancer screening, Salkeld et al. (2016) concluded that personalizing a PDA has a significant impact on men's opinions; the reasoning underpinning these opinions; and on the congruence between opinions and behavioral intentions. Consequently, they suggested that personalized PDAs might be better tools for facilitating communication and decision making than regular PDAs. To date, none of the PDAs for prostate cancer treatment were personalized in terms of outcome probabilities (Vromans et al., 2019). Only recently, Bagshaw et al. (2021) developed a personalized PDA with toxicity tables for the most common side effects, relating pre-treatment health state to the probability of a certain post-treatment health state for different radiation modalities. This PDA, in which men can also incorporate preference thresholds, assign value and evaluate alternative treatments, is currently evaluated in practice.

In the Netherlands, there are several non-personalized PDAs available for localized prostate cancer treatment (Al-Itejawi et al., 2016; Cuypers et al., 2019a), among them are two web-based PDAs developed by Maastro Clinic (Ankolekar et al., 2019) and PATIENT+ (PATIENT+, 2020; van Tol-Geerdink et al., 2013). In the current project, we merged and upgraded the content of these two PDAs by following user centered design principles; taking into account IPDAS criteria; and including (understandable information about) general and personalized risks. In an effort to elicit requirements and ensure optimal understanding and acceptance, different groups of experts and the general public were involved. This paper describes the development process and reports on content, visual and organizational requirements of this personalized PDA for localized prostate cancer treatment.

## 2 Methods

### 2.1 Design

**Fig. 1:**
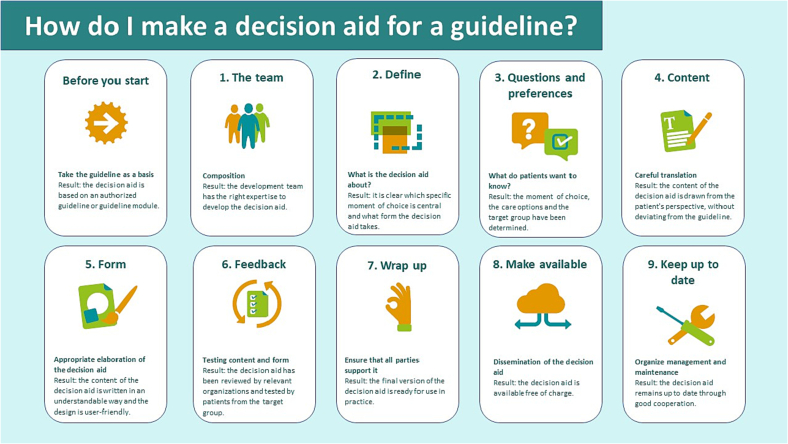
Steps that were followed during the development process, based on the Dutch guide “How do I make a decision aid for a guideline?” (Dutch Association of Medical Specialists).

The development process (January – September 2020) was based on a Dutch guide that provided minimal steps and criteria for the setup of a PDA next to a practice guideline (Fig. 1) (Dutch Association of Medical Specialists). This guide resulted from a scientific exploration including (inter)national sources about the content and format of PDAs (Coulter et al., 2013; Elwyn et al., 2006; Elwyn et al., 2009; Stacey et al., 2017). The Dutch national practice guideline “Prostate cancer” (Netherlands Comprehensive Cancer Organization) was starting point and key principles of user centered design were followed throughout the process (Gulliksen et al., 2003; van Gemert-Pijnen et al., 2011). Ethical approval was obtained from the Medical Ethical Committee Zuyderland Zuyd (METCZ20190155) and participants provided informed consent.

### 2.2 Context

A university of applied sciences, an institute for radiation therapy, a provider of PDAs, and two hospitals collaborated in the PROSPECT (prostate cancer decision aid for side effects) project. Deciding upon the most appropriate treatment (RP, EBRT, BT or AS) for an individual patient after being diagnosed with localized prostate cancer was the focus of this PDA. Other PDA characteristics that were formulated in advance:

• an online tool based on the textual content of two existing PDAs;
• that included personalized risks on side effects based on CPMs;
• that was introduced to the patient by the urologist or nurse after diagnosis;
• that could be used by the patient at home to reconsider options;
• of which the results were then discussed in consultation with the urologist.

### 2.3 Participants

The development team consisted of three researchers (two based at a university of applied sciences and one based at an institute for radiation therapy) and one expert (based at a provider of PDAs) with expertise in development, evaluation and implementation of eHealth interventions in general and PDAs (including artificial intelligence) in particular. Team meetings occurred biweekly. Decision making about (requirements for) content, visualization and organization during team meetings was documented in minutes. Depending on the input needed, various groups of experts were involved during the development process as well as the general public (Table 1). For this purpose, both purposive and convenience sampling were used. Cancer survivors with variation in age, time since diagnosis and educational level were recruited via the prostate cancer patient organization. Health professionals from different disciplines and care organizations as well as usability experts were invited via the formal network of team members. Linguistic experts were contacted via one of the PDA providers. Recruitment of men from the general public occurred via a citizen participation initiative and via the informal network of team members.

**Table 1:** Groups of experts that were involved during the development process.

Group	Participants
Experts by experience	
Group 1a	3 cancer survivors, aged 65–72 years, high educated, diagnosed with prostate cancer between the years 2009–2018, all being representatives from the prostate cancer patient organization
Group 1b	5 cancer survivors, aged 64–85 years, low-high educated, diagnosed with prostate cancer between the years 2000–2020

Professional experts	
Group 2a	5 health professionals (4 urologists and 1 radiotherapist) and 1 researcher (who was engaged in previous prostate cancer PDA development)
Group 2b	3 usability experts with experience in performing different formats for usability testing of online programs
Group 2c	2 linguistic experts that give advice and award certificates regarding easy reading
Group 2d	12 health professionals (3 urologists, 3 nurse practitioners, 3 radiotherapists and 3 general practitioners)

General public	
Group 3a	58 men, aged 58–90 years, high educated, with and without (history of) prostate cancer
Group 3b	5 volunteers, aged 24–32 years, high educated, without (history of) prostate cancer

### 2.4 Procedure

Step 1 and 2 (the team and define) were already set at the beginning of the project. Step 3 (questions and preferences) was initially covered by making use of two existing PDAs, that were developed together with (advocates of) patients and health professionals. As regards step 8 (make available), partnerships of urologists were pro-actively approached to increase awareness. Concerning step 9 (keep up to date), periodic maintenance was arranged with the PDA provider upon completion of the project. Therefore, step 4–7 (content, form, feedback and wrap up) were iteratively worked through and described following the actual process.

### 2.5 Data collection and analyses

Because of COVID-19 and related measures, most activities were performed online with software that complied with national privacy legislation.

#### 2.5.1 Content requirements

##### 2.5.1.1 Content

The textual contents of the two existing PDAs (Ankolekar et al., 2019; PATIENT+, 2020; van Tol-Geerdink et al., 2013) were merged. Alongside this process, survivors (group 1a) were consulted with specific questions about preferences regarding the time frame of side effects (i.e. one or two year after diagnosis) and proxies of side effects (i.e. reliability or stiffness of an erection). Responses were collected via email, stored in a log file and summarized. Moreover, CPMs were created based on static national data from ProZIB (Vernooij et al., 2020) in order to predict personalized risks on side effects. Key information on these CPMs is provided in Box 1, though more details are published elsewhere (Hasannejadasl et al., 2022a, Hasannejadasl et al., 2022b).

Box 1 **Development and external validation of CPMs for erection and urinary problems.**

**Table:** 

CPMs for treatment side effects were based on data from ProZIB (2014–2019), an initiative from the Netherlands Comprehensive Cancer Organization, the professional group for urologists and the prostate cancer patient organization. The database contains demographic information, clinical data and PROMS of patients diagnosed with prostate cancer on three different points in time (upon diagnosis, after one year, after two years). For creating CPMs, the answers on the Expanded Prostate Cancer Index Composite (EPIC26) of 964 patients from 69 hospitals were used. Logistic regression with Recursive Feature Elimination (RFE) was applied and sensitivity, specificity and overall accuracy were calculated. For erection problems, the one-year model required 10 variables (sensitivity 87.1 %; specificity 66.2 %; overall accuracy 75.3 %; AUC 0.84) and the two-year model 9 variables (sensitivity 87.4 %; specificity 62.1 %; overall accuracy 73.7 %; AUC 0.84). Regarding urinary problems, the one-year model was based on 9 variables (sensitivity 82.0 %; specificity 65.1 %; overall accuracy 76.0 %; AUC 0.80) and the two-year model on 9 variables (sensitivity 72.0 %; specificity 40.0 %; overall accuracy 76.0 %; AUC 0.62). Some variables were overlapping; others were different in the one-year and two-year models.

Alt-text: Box 1

##### 2.5.1.2 Feedback

The textual content of the PDA was shared with professional experts (group 2a) in order to receive feedback on correctness and completeness of information. Feedback was collected via email, stored in a log file and summarized. Based on their individual reactions, textual adjustments were made and a web-based prototype was created.

#### 2.5.2 Visual requirements

##### 2.5.2.1 Form

**Fig. 2:**
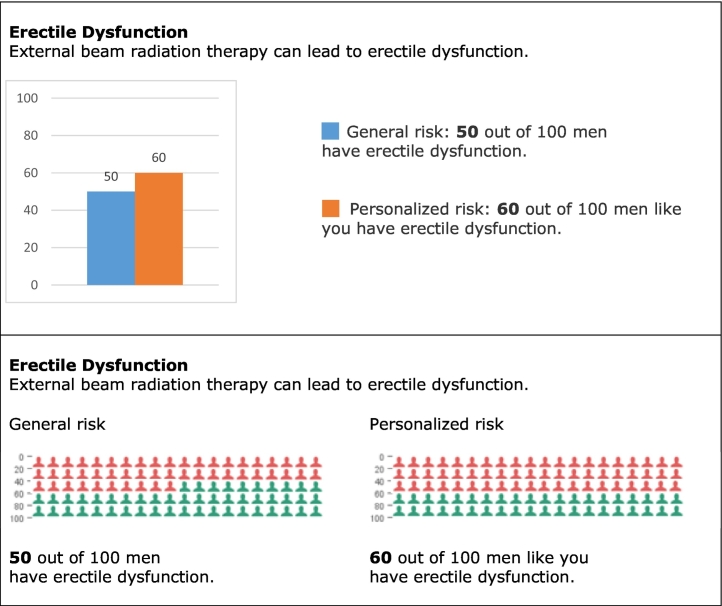
Mock-ups containing different visualizations of risks.

Because evidence about the most effective form of risk communication in case of general and/or personalized risks was lacking, a questionnaire was designed and sent out to the general public (group 3a) via online questionnaire software of Questback (Questback, 2019). Questions were posed about understanding and preferences of risk communication. Risks were presented with words (rarely - sometimes – often - very often); numbers (natural frequencies and percentages); and graphs (icon arrays); either alone or combined. Responses were analyzed by using frequency distributions as well as descriptive statistics in SPSS (IBM Corp., 2020). Outcomes were used by the team to design two mock-ups for usability testing, making use of either icon arrays or bar charts (Fig. 2). Each mock-up contained three visualization: one with general risks, one with general and personalized risks side by side, and one with general and personalized risks via a drop down menu.

##### 2.5.2.2 Feedback

Three usability experts (group 2b) were given an introduction into the topic and a scenario (imagine to be a 60-year old man with prostate cancer who has to decide on treatment supported by the PDA). Based on this scenario, the experts individually performed a heuristic evaluation to assess usability of the prototype and mock-ups in the light of the ten usability principles of Nielsen (2005) and the behavioral patterns of Tidwell (2010). After discussion and consensus, outcomes were used by the team to draft the tasks for subsequent tests. Moderated usability testing with cancer survivors (group 1a) involved one-on-one sessions with a think-aloud walkthrough of the prototype and mock-ups and specific tasks in between. Tasks included, for instance, searching for certain disease related information, comparing risks for different options, and saving and sharing results of the PDA. Throughout testing, notes were taken and Morae software was used for screen and audio recording (TechSmith, n.d.). Recordings were analyzed with deductive coding, (i.e. observation, comment, error and user needs help). For one-on-one qualitative eye-tracking testing, five healthy volunteers (group 3b) were given the same scenario as the experts, went through the PDA and the mock-ups while eye tracking took place, and performed the same specific tasks as the cancer survivors. Notes were taken and recordings were created with eye tracking software from Tobii (Tobii Eyetracking, 2020). Afterwards, volunteers reviewed these recording in fast speed while thinking-aloud. Noticeable eye-movements (i.e. quicker, more and/or longer fixations) were discussed. For the PDA, content was dynamic and data were analyzed qualitatively to find focus areas, neglected areas and patterns in eye-movement. For the mock-ups, content was static and data were analyzed quantitatively with descriptive statistics. Thereafter, the PDA was shared with linguistic experts (group 2c) to receive feedback on the verbal and visual presentation of information. Responses were collected via email, stored in a log file and summarized. Based on all results, visual adjustments were made.

#### 2.5.3 Organizational requirements

##### 2.5.3.1 Wrap up

To better understand implications of the PDA when used in practice from both a health professionals' and patients' perspective, individual semi-structured interviews were held with five cancer survivors (group 1b) and twelve health professionals (group 2d) via Zoom video conferencing software (Zoom Video Communications, 2020). The topics that were discussed with cancer survivors included experiences with SDM; opinions regarding proposed PDA; added value of proposed PDA to SDM consultation; and ideas about PDA implementation. The topic list for health professionals covered the use of current PDAs during SDM consultation; opinions regarding personalized risk information and the proposed PDA; requirements for the proposed PDA; and PDA implementation. All interviews were transcribed at verbatim and analyzed in NVivo (NVivo QSR International Pty Ltd., 2018), following a directed content analysis (Hsieh and Shannon, 2005).

##### 2.5.3.2 Feedback

The prototype was shared with health professionals (group 2a) together with a rationale for decisions concerning textual and visual content and integration into daily practice. Feedback was collected via email, stored in a log file and summarized. Based on their individual reactions, final adjustments were made and ideas about integration into daily practice were summarized. The final PDA was checked by two team members independently using the IPDAS criteria (Elwyn et al., 2009). During a consensus meeting, ratings were compared and differing opinions were discussed and resolved.

## 3 Results

### 3.1 Content requirements

##### 3.1.1.1 Content

As the textual contents of two existing PDAs were merged, variation in naming, definition and figures was revealed. Repeated discussions with the team resulted in consensus about the outline of the PDA, in which some elements were adopted from one of both PDAs and others were new. Following a classification in low, medium and high risk, the PDA included five sections (Box 2). Consultation of two cancer survivors (group 1a) provided confirmation for the ideas to use a one-year time frame consistently and to choose reliability of an erection, urine loss and urgency of bowel movement as proxies for erection, urinary and intestinal problems respectively.

Box 2 **Five sections included in the PDA.**

**Table:** 

• ‘Information’ to educate about localized prostate cancer and procedures, (dis)advantages and side effects of different treatment options; • ‘Comparison’ in an interactive option grid to present all treatment options side by side in terms of procedure, duration, cure, death, (dis)advantages, and side effects; • ‘Key points’ with questions to check understanding of the information that was provided; • ‘Your preference’ to clarify expectations of treatment and to indicate what is really important making use of statements; • ‘Closure’ to verify whether patients feel sufficiently informed to participate in shared decision making.

Alt-text: Box 2

The CPMs for erection and urinary problems after one year turned out to be statistically accurate enough for clinical application; for intestinal problems this was not the case. For that reason, the team proposed to use personalized risks for erection and urinary problems and a general risk for intestinal problems.

##### 3.1.1.2 Feedback

Sharing the textual content with health professionals (group 2a) resulted in feedback on correctness and completeness of information. General suggestions were made for more clear definitions regarding disease classification (i.e. cut-off values for Gleason score, PSA and T-status in low, medium and high risk) as well as side effects (i.e. use of broad terms to enable comparison and avoid words like ‘severe’). For the different treatment modalities, additions were proposed in terms of duration (e.g. hospital stay after RP; EBRT through day treatment), technical details (e.g. use of a robot for RP, procedure for EBRT) and short- and long-term consequences (e.g. temporary catheter and remain at rest, treatment options after initial treatment). Regarding side effects, health professionals extended the list with specific side effects per treatment (e.g. impotence and depressive symptoms along HT) and suggested to be more specific about the nature of side effects for each treatment (e.g. stress incontinence in RP, urge incontinence in EBRT/BT).

### 3.2 Visual requirements

#### 3.2.1 Form

The questionnaire informed about understanding and preferences of general and personalized risks among the general public (group 3a). Men found it difficult to combine information, especially when integrated into one graph (Table 2). Participants mostly chose numbers, alone or together with words or icon arrays; for both general and personalized risks.

**Table 2:** Number of participants (*N* = 58) that answered correctly or provided certain preferences for different forms of risk communication.

Understanding general and personalized risks in graphs	
Reading 2 graphs with separate information	58.6 %
Reading 1 graphs with combined information	27.6 %

Preferences for presenting general and personalized risks	
Words	12.1 %
Numbers	38.0 %
Graphs	3.4 %
Both words and numbers	29.3 %
Both graphs and numbers	17.2 %

Preferences for general or personalized risks	
Only general risks	10.3 %
Only personalized risks	20.7 %
Both general and personalized risks	69.0 %

#### 3.2.2 Feedback

With concern to the PDA prototype, usability experts (group 2b) identified usability problems for three major themes: information structure, navigation, and visual design. Recommendations for information structure included to add an introduction that explains users to go through five steps and include a prominent and attractive call-to-action button in order to start the PDA. To solve navigation problems, the experts advised to be consistent in the use of buttons and to change colors in the menu when parts have been visited. Regarding visual design, the visual hierarchy including composition of the webpage, distribution and alignment of elements, colors and typography needed improvement. In addition, three patients (group 1a) experienced navigation problems and difficulties in visualization and asked for more clarification on terminology. Eye-tracking data from five healthy volunteers (group 3b) confirmed some navigation and visualization problems. Regarding the mock-ups, usability experts (group 2b) preferred either bar charts to describe proportions, quantities or differences, or icon arrays as users may find them easier to identify with. When it comes to the visualization of both general and personalized risks on side effects, placing them next to each other was recommended over drop-down menus. Moreover, words or numbers combined with visualizations could work well, whereas words or numbers by themselves were argued as weak. In addition, a clear coloring scheme was recommended as well as the use of annotations, interactions or help-functions. The linguistic experts (group 2c) advised mainly on how to introduce CPMs and personalized risks (risks for “men like you”) in the PDA and on how to formulate the questions that were used to gather input for the CPM.

### 3.3 Organizational requirements

#### 3.3.1 Wrap up

Although enthusiastic about the personalized PDA, all health professionals (group 2d) stressed the importance of properly discussing the risks of side effects together with patients, even more because patients differ in numeracy and graph literacy. Urologists acknowledged to have little time for SDM, but improvements were seen since the introduction of case managers or nurse practitioners. Both general practitioners and radiotherapists expressed a desire to be more involved in this process. None of the cancer survivors (group 1b) made use of a PDA, though patients preferring an active role in decision making confirmed that the personalized PDA would have been helpful and even could have resulted in a different decision. Patients suggested taking the proposed PDA home after diagnosis, so that information could be reviewed in peace and quiet, together with beloved ones.

#### 3.3.2 Feedback

**Fig. 3:**
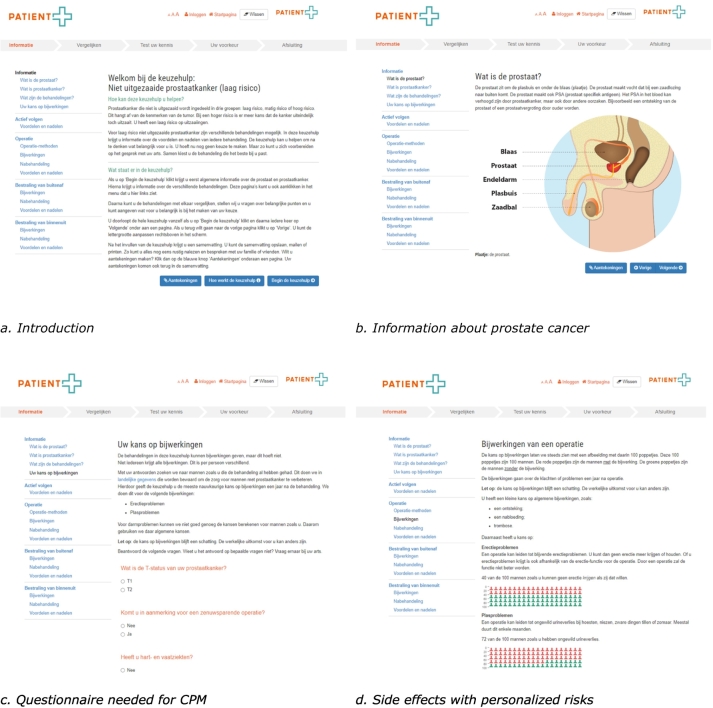
First impression of the final PDA (in Dutch).

Asking professional experts (group 2a) for their ideas about use of the PDA in daily practice, highlighted some important issues that needed consideration. Experts agreed on the proposed process of introducing the PDA to patients during the consultation in which the diagnosis is disclosed, incorporating personalized risks (when available) during a multidisciplinary meeting, and discussing all the results of the PDA during the consultation in which a treatment decision is made. At the same time, experts wanted to know when, how, and by whom personalized risks were to be included in the PDA, especially because the information needed for these risks could not yet be extracted from electronic patient records. Moreover, experts mentioned that risks - though personalized - remain risks. When introducing the PDA to patients, not only personalized risks, but also related uncertainties needed to be explained very carefully. For that matter, experts proposed to base general and personalized risks on the same database as well as to present either a personalized or general risk for a certain side effect. Based on the IPDAS checklist, the final version of the PDA still showed room for improvement. Negative and positive features of options were mentioned, though not in all five sections (Information - item 8); the time period and uncertainties of outcome probabilities were not specified (Probabilities - item 4, 6); the psychological and social impact was lacking (Values - items 2 and 3); the PDA was not reviewed by patients not involved during development nor tested by patients facing the decision or practitioners that counsel these patients (Development - items 3, 5, 6); quality of evidence was not described (Evidence - item 5); contributors were not mentioned clearly (Disclosure and transparency - item 2) and evidence about the added value in practice was lacking (DST evaluation - item 1, 2). Based on the entire development process, a final version of the PDA was made ready for use in practice (Box 3, Fig. 3).

Box 3 **Description of a first final version of the PDA.**

**Table:** 

Regarding the content, the PDA was developed for low, mediate and high risk localized prostate cancer and included information about four treatment modalities (RP, EBRT, BT, and AS) and three main categories of side effects (erection, urinary, and intestinal problems). Information about the different procedures, (dis)advantages and side effects was presented in five sections that educated patients, enabled comparison, checked for understanding, clarified preferences, and verified readiness for SDM. In the first section (‘information’) an explanation was added about the meaning of risks for ‘men like you’ as well as the 16 questions that related to the variables needed to calculate these personalized risks for erection and urinary problems one year after diagnosis. Because the CPM for intestinal problems did not meet expectations, a general risk was included. All risks, both general and personalized, were derived from the same dataset. With concern to visualization, three icon arrays were included for each treatment modality in the ‘information’ section, one for each side effects. The icons arrays were accompanied with an explanation about the 100 icons representing 100 “men like you” and the colors indicating the presence or absence of a particular side effect. Eventually, only a general or personalized risks was depicted, to avoid unnecessary complexity and misunderstanding.

Alt-text: Box 3

## 4 Discussion

Because various treatment options are comparable in outcomes in terms of survival and recurrence, though different in risk as well as severity and nature when it comes to side effects, treatment decisions for localized prostate cancer are highly preference-sensitive. To better inform patients and support SDM, the development of a PDA including personalized risk information for side effects was proposed. This paper reports on requirements in terms of content of information, visualization of risk profiles, and use in practice as a result of an iterative and co-creative development process.

Content requirements were defined based on the practice guideline, the content of two existing PDAs, repeated discussion with the team and feedback of professional experts. Content-wise the PDA needed to differentiate between risk groups; to include information about the most common treatment modalities and the main categories of side effects; and provide explanation about the meaning and uncertainties of (personalized) risks. In further establishing the details of these requirements, patients with localized prostate cancer seem to consider whether treatment options could eliminate their cancer, extend their survival, delay disease progression and preserve quality of life considering treatment side effects (Zeliadt et al., 2006). In addition to providing information and improving knowledge, patient empowerment needs to be supported. PDAs that only focus on providing information fail to address that a preference sensitive decision needs to be made for which patients involvement is key (Joseph-Williams et al., 2014). Lack of awareness in this respect is an important patient barrier for SDM (Martínez-González et al., 2018).

Visual requirements were drawn from an exploration among the general public and a more in-depth analysis with usability experts, cancer survivors and healthy volunteers. Visual aids to communicate statistical information and personalized outcome predictions, so-called communicative aspects, could further improve PDAs for prostate cancer treatment (Vromans et al., 2019). With concern to the visual design, the PDA needed to visualize risks accurately for which bar charts or icon arrays were recommended; to choose a clear coloring scheme consistently throughout the PDA; to complement visualizations with words or numbers and a clear explanation or legend; and to present risks next to each other to facilitate comparison. A combination of verbal descriptors and numerical estimates is advised both within PDAs and during consultations in order to avoid an overestimation of personalized risks for side effects (Vromans et al., 2020). Moreover, incorporating comparative risk information for personalized screening PDAs has shown unintended results and therefore needs to be contemplated carefully (Fagerlin et al., 2007). To illustrate, when personalized risks are below average, comparative information may discourage patients from undergoing treatment that otherwise might have been chosen. At the same time, when personalized risks are above average, patients might be inclined to undergo treatment that otherwise might not have been chosen.

Information about organizational requirements was gathered during interviews with health professionals that counsel patients with localized prostate cancer facing a treatment decision and cancer survivors themselves. Before actual use in practice, the PDA needed to be integrated into local clinical pathways. Moreover, the importance of clear agreements were mentioned about input and output of information in terms of whom, what and when. Previous research informs about a common PDA workflow: the diagnosis and possible treatments are discussed in an oncology meeting together with the suitability of handing out a PDA; a nurse prepares the invitation for the PDA; the patient receives the diagnosis and the PDA is provided during a consultation by the urologist or during a follow-up consultation by the nurse; the patient returns for the treatment decision and discusses the outcomes of the PDA during a consultation with the urologist (Cuypers et al., 2019b). When present, close involvement of nurse practitioners was recommended here, particularly because the meaning of (personalized) risks and related uncertainties needs to be explained carefully, taking into account differences in numeracy and graph literacy. Studies emphasized the key role of nurses in supporting men making prostate cancer treatment decisions by providing PDAs, answering questions, and advocating preferences (Stacey et al., 2020). The involvement of nurses is appreciated by patients as well (van Tol-Geerdink et al., 2020) and believed to positively affect PDA uptake (Cuypers et al., 2019b). Some lessons learned from the development process are summarized in Table 3.

**Table 3:** Lessons learned during the development of a PDA with personalized risks.

Approach	A step-by-step guide is useful to structure the development process. The iterative character with frequent moments of expert feedback makes it possible to make timely adjustments. The involvement of different groups for different parts of the PDA is valuable.
Stakeholders	Before the project kick-off, serious thought needs to be given to relevant stakeholders, also in terms of interest and influence. Interdisciplinary collaboration requires attention, as different stakeholders speak different languages. Early involvement of patients and health professionals as actual members of the development team is important to focus on the user perspective.
Content	The project needs to start with conceptualizing, with respect to side effects, suitable proxies and relevant time frames. During data collection, not everybody feels comfortable to talk about side effects of prostate cancer.
Visuals	As patients differ in both preferences as well as numeracy and graphical literacy, communication about personalized risks might best be done with a combination of formats. Patients need to be explained the meaning of a personalized risk, being personalized yet still a risk. The complexity of risk communication further increases when only part of the risks can be personalized.
Organization	Aside from content and visual requirements, organizational requirements are just as important in order to implement PDAs in practice. Health organizations differ in their organization of prostate cancer treatment, in terms of when, how, and by whom; the PDA needs to be aligned with local care paths. Health professionals need to understand outcomes and uncertainties of CPMs and be able to explain them to patients.

Some strengths and limitations of the development process need to be addressed. First, for the personalized PDA the national practice guideline was starting point together with two existing PDAs. Integrating the latest evidence as well as not starting from scratch, initially made things easier. At the same time, the chosen platform gradually introduced some challenges in responding to needs and wishes from experts, because of technical, budget and/or time constraints. Second, extensive usability testing largely impacted the information structure, navigation, and visual design of the PDA. Unfortunately, highly educated patients and volunteers were overrepresented. Third, a step-by-step guide was used to structure the development process. As this guide mainly informed about the content of the PDA, and not so much about visuals and organization, the process was somewhat extended. For this purpose, the involvement of different expert groups and the general public was important and useful. Aside from consultation, various other forms of involvement could have been even more valuable, especially for patient participation (de Wit et al., 2011; Shah and Robinson, 2007).

In addition to the requirements, the IPDAS checklist provides further guidance for finalizing the PDA. Some of the IPDAS items, such as balanced information about pros and cons and time periods throughout the entire PDA, still need to be processed during the development whereas other improvement measures can be tackled thereafter, including testing the PDA with patients and health professionals during actual SDM and gather evidence about the added value in practice. For this purpose, implementation and validation studies are prepared. In parallel, effort is needed to increase knowledge and awareness about the use of CPMs in PDAs in order to overcome resistance. Aside from a lack of training, resources and time, limited confidence of health professionals in CPMs seems an important barrier for successful implementation of PDAs (G. Elwyn et al., 2013; Légaré et al., 2008). Apart from that, sustainability of data is an important issue. CPMs based on static data can become outdated and consequently no longer accurate (Jenkins et al., 2021; Jenkins et al., 2018). PDAs need to be updated periodically, for which a dynamic or living database including new data about conventional as well as experimental treatments is conditional.

## 5 Conclusions

To our knowledge, this is the first Dutch PDA for patients with localized prostate cancer that includes CPMs to estimate risks of treatment side effects. Guidelines for both content and process guided the development and resulted in content, visual and organizational requirements that are translated into the final version of the PDA. Co-creation with various groups of experts and the general public brought in different perspectives, intended to ensure optimal alignment of the PDA with potential users and the situation in practice.

## Declaration of competing interest

The authors declare that they have no known competing financial interests or personal relationships that could have appeared to influence the work reported in this paper.
